# Impact of land use type conversion on carbon storage in terrestrial ecosystems of China: A spatial-temporal perspective

**DOI:** 10.1038/srep10233

**Published:** 2015-05-15

**Authors:** Mei Zhang, Xianjin Huang, Xiaowei Chuai, Hong Yang, Li Lai, Junzhong Tan

**Affiliations:** 1 School of Geographic & Oceanographic Sciences, Nanjing University, Nanjing 210023, Jiangsu Province, China; 2 School of Urban and Resources Sciences, Jinling College of Nanjing University, Nanjing 210089, Jiangsu Province, China; 3 Centre for Ecological and Evolutionary Synthesis, Department of Biosciences, University of Oslo, Blindern 0316, Oslo, Norway; 4 Jiangsu Information Center, Nanjing 210013, Jiangsu Province, China

## Abstract

Our work is the first study to explore the national and provincial composite carbon storage variations in terrestrial ecosystems of China caused by the entire flows of land use type conversion (LUTC). Only water body was excluded. The results indicated that terrestrial ecosystems of China lost 219 Tg-C due to LUTC from 1980 to 1995, and the amount was 60 Tg-C during the period 1995-2010. Despite the decrease in the total amount, carbon losses from LUTC intensified, but most of the losses were balanced by the opposite conversions. Our analyses also revealed that LUTCs in China were becoming detrimental to carbon reduction, mainly due to the insufficient increase of forest land to meet the growing demand for carbon absorption, the accelerating disappearance of grassland and the rapid expansion of settlements. More than 50% of the carbon storage variations for a single LUTC flow concentrated in several provinces. To improve China’s LUTC status from the aspect of low-carbon, Heilongjiang, Sichuan, Inner Mongolia, Tibet, Qinghai, Xinjiang and coastal regions, such as Shandong, Jiangsu and Liaoning, should be dealt with first according to their conditions. This study can be helpful to planners, policy makers and scholars concerned about carbon reduction in China.

This paper analyses how the national and provincial flows of land use type conversions (LUTCs) have influenced carbon storage in the terrestrial ecosystems of China during recent decades. Land use change is a major driver to carbon storage variation in terrestrial ecosystems^1^,^2^. The most intense part of land use change, LUTC, can cause drastic carbon storage changes. LUTC leads to changes of vegetation, which directly influence vegetation carbon storage^3^. For example, when forest land, which usually has the highest amount of vegetation biomass^4^, is converted to another land use type, it releases carbon into the atmosphere in addition to the parts reserved in products, such as the furniture made of the harvested timber^5^,^6^. Furthermore, the vegetation changes caused by LUTC influence the vegetation residue returned to soil, together with other influences such as soil cultivation or disturbance, and therefore LUTC leads to the changes of soil organic carbon (SOC) storage^7^,^8^,^9^. In a word, vegetation carbon and SOC storages of terrestrial ecosystems vary when LUTC occurs. Terrestrial ecosystems play important roles in reducing carbon emission and offsetting global warming, which has been the subject of worldwide concern in recent years^10^,^11^,^12^,^13^. With its continued or intensified activities, LUTC has been noticed for its important influence on carbon storage in terrestrial ecosystems^1^,^14^,^15^,^16^.

To date, extensive research has been done on land use related carbon storage variation in terrestrial ecosystems^17^,^18^,^19^. However, a large part of the literature focused on the carbon sink capacity of terrestrial ecosystems while maintaining the land use types^20^,^21^,^22^,^23^. And studies of LUTC related carbon variations generally focused on single terrestrial ecosystems or limited land use types^24^,^25^. In addition, due to factors such as vegetation type, soil type and LUTC intensity, carbon storage variations caused by LUTC differ between regions^6^. However, analyses on distributions of carbon storage variations from LUTC are very limited. With the accelerating development and the increasing pressure to reduce carbon emission and air pollution^26^,^27^, China has become an important study area. However, national studies have only focused on the total amount or on the general divisions, while provincial studies have only considered the single province^15^,^28^,^29^,^30^,^31^. To our knowledge, no comparisons have been made between the provincial divisions of China concerning the carbon storage variations of terrestrial ecosystems caused by the entire flows of LUTC. What are the statuses of the national and provincial LUTC flows in China from the aspect of carbon storage variation? Is the situation beneficial for carbon reduction in China? Which provincial regions have the greatest carbon storage variations? What is the trend of carbon storage variations? All these questions remain unanswered. Because national and provincial units share most of the responsibility for reducing carbon emissions in China, analyses at the provincial scale across the entire China can be very helpful, especially given the ongoing new-style urbanisation movement^32^.

In this paper, we used Remote Sensing (RS) and Geographical Information Systems (GIS) technologies to estimate carbon storage variations from LUTC. To better understand the trend of carbon storage variations, the years from 1980 to 2010 were divided into two periods, 1980-1995 and 1995-2010. Using land use data, a provincial zoning map, and data related to vegetation carbon and SOC densities, this study investigated the national and provincial flows of LUTC in China, calculated the provincial carbon densities of different land use types, and estimated and analysed the national and provincial carbon storage changes caused by LUTC. The implications of the results are discussed, and suggestions for future land use are made. The provincial regions studied in this paper are shown in Fig. 1.

## Results

### The flows of LUTC from 1980 to 2010

**Figure 2:**
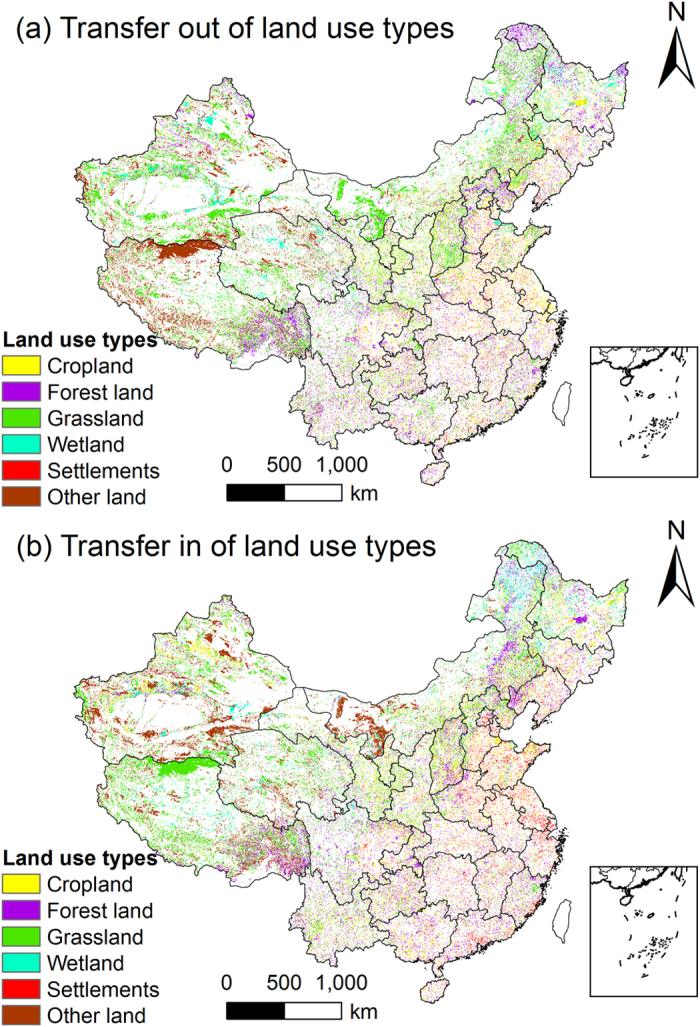
The flows of LUTC from 1995 to 2010. Map created using ArcGIS 9.3 software.

LUTC activities in China were considerable during these three decades. From 1980 to 1995, LUTC activities covered approximately 15.1% of China’s area, and the percentage increased to 22.3% during the period 1995-2010. We mapped the LUTCs of the later and more intense period for the six analysed land use types (Fig. 2). Analyses revealed that grassland suffered the greatest change. Grassland transferred out from 1980 to 1995 was 5.28 × 10^7^ ha, while only 4.71 × 10^7^ ha was turned into this land use type. During the next 15 years, the area lost increased to 7.71 × 10^7^ ha, while the area gained was only 6.12 × 10^7^ ha. Therefore, China consistently lost grassland during these 30 years, and the process was accelerating. During the first 15 years, this loss was concentrated in Western China, particularly in Tibet. However, the most affected regions shifted to Northwest China in the following 15 years, with Inner Mongolia and Xinjiang as the top regions. The most important cause for the loss of grassland was desertification. Although the losses of forest land and cropland were also significant, China managed to keep these two land use types in balance, mainly because of the lasting attention paid to deforestation and food production. We found that forestation was the second reason for the loss of grassland. The changes of wetland were not intense in any region except Xinjiang, Inner Mongolia, Heilongjiang, Qinghai and Tibet. It was also worth noting that settlements expanded quickly as China developed, particularly in the coastal regions, such as Shandong, Jiangsu and Liaoning. The area that was converted to settlements nearly doubled during the period 1995-2010 compared with the earlier 15 years. From 1980 to 1995, China was able to control the area of settlements by converting unimportant or wasted settlements to other land use types. However, this became more difficult to control in the following years.

### Carbon densities of different land use types

**Figure 3:**
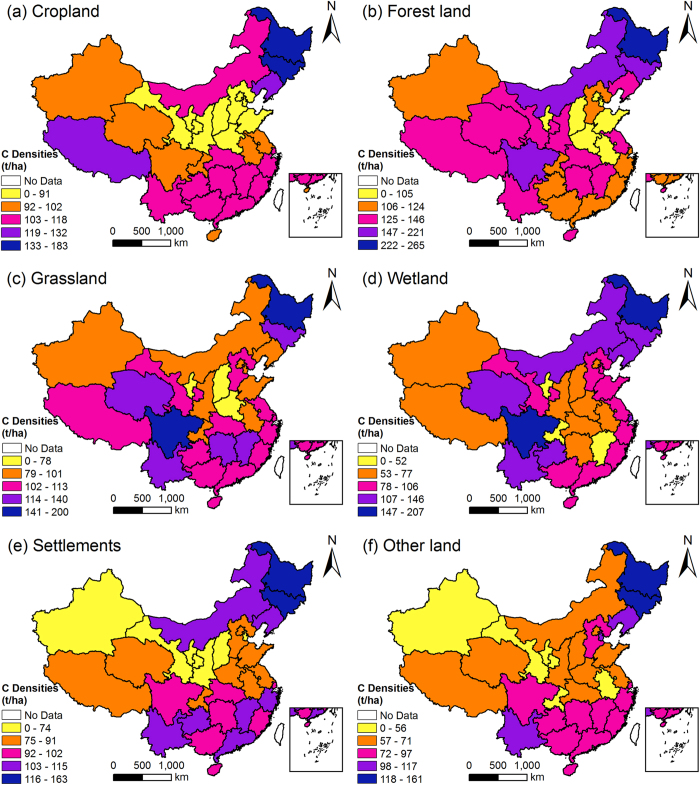
Distributions of composite carbon densities of different land use types. Map created using ArcGIS 9.3 software.

According to our analyses, SOC densities were much higher than vegetation carbon densities for all land use types in any region. By comparing the average values of the same land use type, we found that the ratio of the two densities ranged from 7.31 to 14.36. The provincial distributions of the two densities differed widely. We only analysed the distributions of composite carbon densities (Fig. 3). Composite carbon densities varied widely between provincial regions and land use types. Geographically, for almost all land use types, regions with the highest carbon densities mainly distributed in Northeast China, followed by Southern China. The lowest carbon densities mainly occurred in Central China. The region with the highest average carbon density was Heilongjiang, while the region with the lowest average carbon density was Ningxia. Despite the similar patterns, the carbon density distribution of each land use type was unique. In each region, carbon densities of different land use types also varied, but the differences were less than those between regions, and patterns can be found here. Among the six land use types, forest land usually had the highest carbon density, settlements and Other land usually had the lowest carbon densities, and the other three land use types had intermediate carbon densities. Wetland had lower carbon densities than cropland or grassland in many regions. The carbon density of cropland was higher than that of grassland in some regions, such as Inner Mongolia and Jilin, while the relationship was opposite in other regions, such as Hebei and Heilongjiang. Settlements had higher carbon densities than Other land in most regions.

### Carbon storage changes caused by LUTC

Using the LUTC and carbon density values, we calculated the carbon storage changes caused by LUTC. As shown in Table 1, from 1980 to 1995, a total of 219 Tg-C was lost from terrestrial ecosystems in China due to LUTC, and the amount lost reduced to 60 Tg-C during the period 1995–2010. Although the total amount decreased, individual carbon storage variations intensified. During the transfer out of land use types, the total effects of forest land and grassland were carbon loss, and the increase rates were 42% and 5%, respectively. During the transfer in of land use types, the total effects of cropland, settlements and Other land were carbon loss, and the increase rates were 49%, 123% and 40%, respectively. Fortunately, most losses were balanced by the opposite conversions. The situation of wetland was different from that of the other land use types. The total effects were both carbon loss for the transfer out and in of wetland from 1995 to 2010, and during the conversions to wetland, the amount of carbon loss increased tremendously compared with the earlier period. Most of the carbon storage changes were due to soil rather than vegetation. Soil and vegetation showed the same carbon loss or gain effect for most of the time, however, sometimes they showed the opposite effects instead.

**Figure 4:**
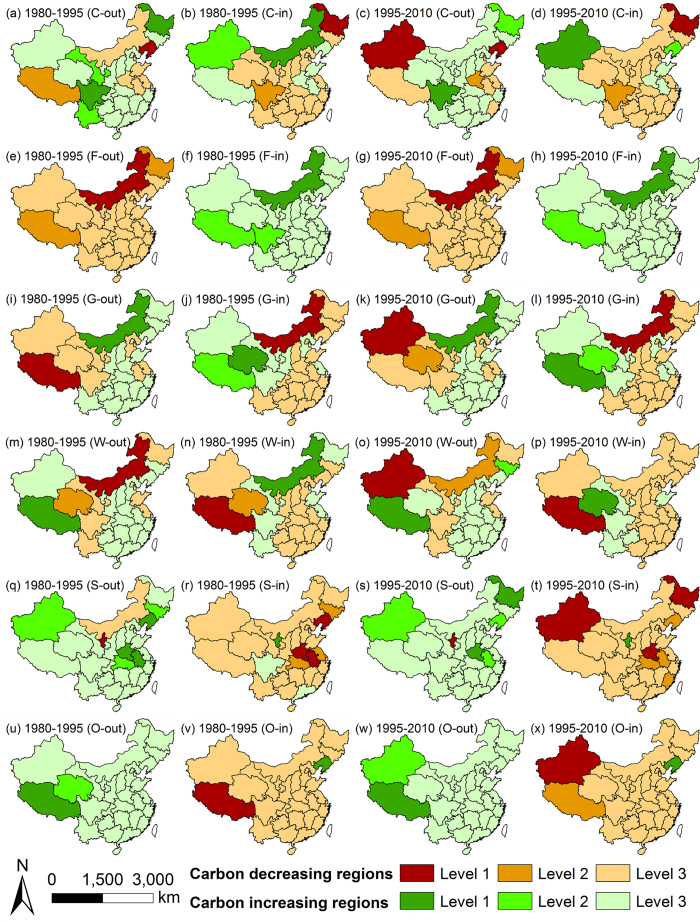
Provincial distributions of carbon storage variations caused by the flows of LUTC (C stands for Cropland; F stands for Forest land; G stands for Grassland; W stands for Wetland; S stands for Settlements; O stands for Other land). Map created using ArcGIS 9.3 software.

By analysing the composite carbon storage changes, we found an interesting phenomenon: for a single LUTC flow (the transfer out or in of a single land use type), over half of the carbon increases or decreases concentrated in a limited number of regions. Based on this characteristic, we divided the regions into groups. First, we grouped the regions as carbon loss or gain regions for a single LUTC flow. If the effect of this change was purely carbon loss or gain, then this step was skipped. Second, taking one type of regions, e.g., the carbon loss regions, we ordered the values in a decreasing sequence and calculated the cumulative percentage for each region. Third, we divided the regions into levels 1, 2 and 3, using 25% and 50% as the breakpoints. Then, regions of the other type were processed similarly. After all LUTCs were addressed, we mapped the results, as shown in Fig. 4. Level 1 and Level 2 regions were the most important regions, therefore, we made Table 2 and Table 3 to show how much they decreased or increased. Values less than 1 Tg-C were ignored.

According to the results, cropland could either lose or gain carbon during a single LUTC activity (Fig. 4a–d). However, the dominant effect for the transfer in of cropland was carbon loss, and the transfer out of cropland mainly caused carbon increase. The most noticeable regions for conversions of cropland were Heilongjiang and Sichuan (Table 2 & Table 3). From 1980 to 1995, these two provinces were the top regions for carbon storage variations during the conversions from or to cropland. This situation continued for the next 15 years. However, some conditions were different for these two regions. Carbon loss and gain were balanced in Sichuan for each period, while Heilongjiang continuously lost carbon storage. Its loss for the two periods were 66 Tg-C and 44 Tg-C, respectively.

The carbon storage variations of forest land were much simpler than those of cropland. The transfer out of forest land only caused carbon loss, and the major responsible regions were Inner Mongolia, Tibet and Heilongjiang (Fig. 4e–g & Table 2). The transfer in of forest land always led to carbon gain, and the most important regions for which were Inner Mongolia and Tibet (Fig. 4f,h & Table 3). Conversions of forest land in Inner Mongolia were becoming beneficial for the environment. During the two periods, Inner Mongolia changed from barely balancing its own carbon loss from deforestation to absorbing 188 Tg more carbon than it lost. However, we should also note that Heilongjiang lost far more carbon than it absorbed during the period studied, which paralleled its situation for cropland.

Similar to the LUTC of cropland, a single LUTC of grassland caused either carbon loss or sequestration, depending on the region (Fig. 4i–l). However, the dominant rule for grassland was similar to that for forest land, and the opposite regions except Inner Mongolia were negligible (Table 2 & Table 3). The results for grassland in Inner Mongolia opposed the dominant pattern, due to the active conversions with forest land and cropland, especially forest land. The regions most noticeably followed the dominant rule were Tibet, Qinghai and Xinjiang. However, situations for the three regions were different. Tibet lost 313 Tg-C from 1980 to 1995, however, in the next 15 years, Tibet gained 315 Tg-C. Qinghai successfully balanced its own carbon decreases during 1980 to 1995, but it caused a bit more carbon loss than gain in the next period. On the other hand, Xinjiang became the new top region for carbon loss in the changes of grassland.

Wetland could cause either carbon decrease or increase during a single LUTC activity, just like cropland and grassland (Fig. 4m–p). However, there were no obvious dominant patterns to be found, which was unique. But it was evident that carbon loss became dominant during the later period (Table 2 & Table 3). The important regions for wetland were the same as those for grassland with the exception of Jilin. The values caused by the LUTC of wetland were less than those of the previously discussed land use types.

The transfer in of settlements mainly led to carbon losses, and the transfer out mainly caused carbon gains (Fig. 4q–t). The values in regions in which the opposite occurred were less than 1 Tg-C. The carbon storage variations were more scattered for settlements than for other land use types, and the amounts were relatively small (Table 2 & Table 3). This result occurred because regions in China were competing with each other in urbanisation, and settlements only took up a small portion of the entire China.

All regions except Liaoning exhibited carbon losses during the transfer in of Other land, and all regions exhibited carbon gains during the transfer out (Fig. 4u–x). The values of Liaoning was less than 1 Tg-C. The major regions were the same as those of grassland, i.e., Tibet, Xinjiang and Qinghai (Table 2 & Table 3). Actually, carbon losses from the transfer out of grassland mainly happened during the conversion of grassland to Other land.

## Discussion

Land use type conversion (LUTC) has important impacts on carbon storage in terrestrial ecosystems^6^,^7^. Therefore, carbon changes from LUTC has been included in the Land Use, Land Use Change and Forestry (LULUCF) sector by the Intergovernmental Panel on Climate Change (IPCC), which has made continuous efforts to provide the policies and suggestions for estimating and controlling carbon emissions^33^,^34^,^35^,^36^. However, studies on LUTC related carbon changes are still very limited in China, mainly due to the complexity of the LUTC process and the lack of available accurate data. In this study, we calculated the composite variations of vegetation carbon and SOC storages. In addition to the widely studied forest land, cropland and grassland^3^,^37^, the flows between all land use types except water body were estimated. Instead of merely considering the general amount or the general divisions^6^,^38^, we made close comparisons between provincial regions, periods, out and in flows of LUTC, and land use types. Due to lack of data, water body was not analysed in this paper, however, this is also a land use type worth studying^39^,^40^,^41^.

Nevertheless, our study did not cover many detailed variations during the carbon changes of LUTC, so the carbon storage variations calculated in this paper should more accurately be called potential changes. The process of LUTC is dynamic and complex. LUTC can take place anytime, but we only tracked the changes of land use types between specific time points. Therefore, the duration of LUTC might be overestimated, and hence the carbon changes might be bigger. In addition, a certain piece of land might have undergone LUTC immediately before the calculation, which will impact the representativeness of the average density used in the calculation of this piece of land. Another fact is that the change of carbon storage in terrestrial ecosystems is a gradual process, which does not happen overnight. The carbon stabilizing cycle should be taken into consideration. The time interval used in this paper, 15 years, is long enough for most types of vegetation to reach the carbon storage level of the converted land use type, but it takes longer for the soil carbon to stabilise^1^. According to Cannell *et al.* (1999)^42^, it may take 25 years to lose 99% of the soil carbon but 100 years to gain 99% in a temperate system. Therefore, this might also lead to overestimation of carbon changes, especially the carbon increases. This might suggest that the efforts on carbon balance in China might have not been performed as well as that revealed in this study. On the other hand, some other researchers have found that SOC densities in terrestrial ecosystems of China were far from the state of saturation, and SOC densities of some land use types increased during recent years^37^,^43^,^44^. In addition, the vegetation carbon density of forest land might have also increased with the growth of trees. Therefore, the carbon storage changes may be underestimated in this paper because these variations were not considered. It is also worth noting that the delineation of wetland might be imperfect because it is an entirely new classification created from the second-level land use types during the reclassification. Although this methodology is preliminary, it easily achieved spatial and temporal comparisons based on unified calculations, which would otherwise require significant amounts of time and resources^3^.

To our knowledge, this is the first attempt to calculate the national and provincial comprehensive carbon storage variations from the entire flows of LUTC in terrestrial ecosystems of China. Direct comparisons cannot be found. The only comparable national estimation can be found in the study of Lai and Huang (2011)^15^. However, carbon changes caused by the LUTC flows were not so fully considered in their study. According to their calculations, China lost 23.7-73.1 Tg-C due to LUTC between 1980 and 2005, which is less than our results. According to our analyses, forest land had the highest carbon density, which is consistent with the studies of Fang *et al.* (2007)^45^ and Zhang *et al.* (2013)^6^. We also found that settlements and Other land had the lowest carbon densities, while cropland and grassland had moderate carbon densities. These patterns are consistent with the research of Chuai *et al.* (2013)^4^. Our work found that China has lost a significant amount of grassland since 1980, which is in agreement with the results of Lai and Huang (2011)^15^. By analysing related studies, Houghton *et al.* (2012)^46^ found that the average annual amount of global carbon emissions from land use and land-cover change (LULCC) was 1.1-1.6 Pg-C yr^−1^. Therefore, from 1980 to 1995, the national carbon storage loss from LUTC estimated in our paper was equal to 0.9%-1.3% of the global carbon emissions from LULCC, and the percentage was reduced to 0.3%-0.4% during 1995 to 2010.

According to our study, although carbon storage losses intensified in the LUTC of each land use type, most of the losses were balanced by contrary conversions due to the efforts of the Chinese government. The total carbon storage loss even decreased during 1995 to 2010. However, further analysis revealed that the status was heading towards the opposite direction of carbon reduction. According to Piao *et al.* (2009)^23^, China’s terrestrial ecosystems absorbed only 28%-37% of the national fossil carbon emissions during the 1980s and 1990s. With the fast development of industry and urbanization, carbon emissions in China are increasing in recent years. According to the relevant literature, carbon emissions from energy consumption in China were 1.56 Pg in 2005^15^, 1.66 Pg in 2006^47^ and 1.78 Pg in 2007^4^. Our study showed that both the national area and the carbon storage of forest land were well balanced during LUTC in the two periods. However, our research also revealed that China lost 2.16 × 10^7^ ha of grassland at an increasing rate between 1980 and 2010. Forestation was the second reason for the rapid decrease of grassland in China besides desertification. Because forest land and grassland are the major carbon sink land use types in China^23^, the carbon sink ability of terrestrial ecosystems in China decreased during LUTC, while the need for carbon absorption increased. Besides these two land use types, settlements should also be noticed. According to our study, the LUTC of settlements caused a total loss of 47 Tg-C during the period studied. However, the rapid expansion of settlement areas caught more of our attentions than the variations in carbon storage. The main carbon emissions of settlements come from its carriers rather than terrestrial ecosystems. The major sources are fossil fuel, transportation, human respiration, livestock, and others^48^. The rapid growth of settlements is consistent with the increase of carbon emissions in China. Therefore, the total situation is challenging for people concerned about carbon emissions in China. To change this situation, great efforts are needed to reverse the total impact of LUTC from carbon loss to carbon absorption.

Our study found that carbon storage changes from LUTC varied widely between the 31 provincial regions, and more than half of the carbon storage variations for a single LUTC flow were due to a small number of regions, as shown in Fig. 4, Table 2 and Table 3. This happened because both carbon densities and LUTC intensities varied widely between regions, as indicated in Fig. 2 and Fig. 3. To improve the LUTC situation in China from the aspect of carbon reduction, we should start with the key regions.

According to our results, the key regions are Heilongjiang, Sichuan, Inner Mongolia, Tibet, Qinghai and Xinjiang. Each region should be treated according to its unique situation revealed in this paper. In Heilongjiang, forest land should be strictly protected or recovered, and the expansion of cropland should be controlled. In Sichuan, the effective measures for compensating carbon loss during the increase of cropland should be encouraged and strengthened, while the rapid increase of cropland should slow down. In Inner Mongolia, the growth of forest land should be encouraged, but desertification should be treated seriously to control the loss of grassland. In Tibet and Qinghai, the effective measures should be continued to treat desertification, and more efforts are needed in Qinghai. In Xinjiang, the rapid desertification should be taken seriously, and successful examples in Tibet and Qinghai should be studied to help solve this problem. In addition, the rapid expansion of settlements in coastal regions should be controlled. As our results showed, the control of expansion became more difficult due to China’s fast development, but efforts should still be made. And compensating measures, such as increasing the areas of carbon sink land use types, planting more trees in settlements, should be strengthened.

In summary, this paper explored the national and provincial flows of LUTC in China from the aspect of carbon storage variations, and discussed the results from the angle of carbon reduction. We found that LUTC activities in China were heading towards the opposite direction of carbon reduction, and the main reasons were the insufficient increase of forest land to meet the growing demand for carbon absorption, the accelerating disappearance of grassland and the rapid expansion of settlements. To improve the LUTC status in China from the aspect of carbon reduction, we recommend beginning with Heilongjiang, Sichuan, Inner Mongolia, Tibet, Qinghai, Xinjiang and the coastal regions, according to their distinctive situations revealed in our work. In China, carbon control plans and policies are mainly carried out on the national and provincial scales. The effective implementation of new environmental law will greatly promote the reduction of carbon emission^49^. Therefore, this study can be very helpful for planners, policy makers and scholars concerned with carbon reduction in China.

## Methods

### Data sources

**Figure 5:**
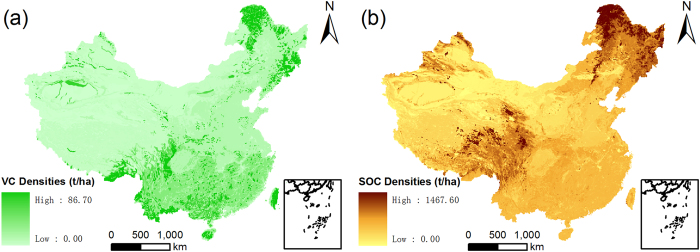
Distribution maps of vegetation carbon (VC) and SOC densities in China. Map created using ArcGIS 9.3 software.

Data used in this research include: a provincial administrative zoning map, three land use maps of China in 1980, 1995 and 2010, and two maps of vegetation carbon and SOC densities in China. The Chinese provincial administrative zoning map was provided by the National Geomatics Center of China. Due to data limitations in Taiwan, Hong Kong and Macao, these areas were excluded in this study. The 1 × 1 km land use raster data of China in 1980, 1995 and 2010 were made by the Institute of Remote Sensing Applications, Chinese Academy of Sciences, using MODIS and Landsat-TM images. Due to the lack of satellite images in 1980, the land use map for 1980 was based on satellite images from the 1980 s, and it is the earliest land use map that can be made using satellite images. This might lead to some variations in the data. The classification system of the land use maps include 6 first-level types, 25 second-level types, and 31 third-level types. To make this study comparable with literature using the IPCC classification system, we reclassified the first-level land use types as cropland, forest land, grassland, wetland, settlements, water body and Other land. Other land includes sandy land, gobi, bare land, rock and gravel, glaciers and firns and other unused land, and its contents vary according to the specific region. We analysed the results using the new first-level land use types. Due to the data limitations of vegetation and SOC density, water body was excluded in this study. To make the distribution map of vegetation carbon densities, we collected data from over 800 related studies, which included almost every type of vegetation found in China^15^. With these data, average carbon densities of 50 different vegetation types in recent years were estimated. Taking the results and the vegetation type map of China compiled in the 1980s^50^, the distribution map of vegetation carbon densities in China was produced using the ArcGIS 9.3 software (Fig. 5a). The distribution map of SOC densities in China was produced using the soil type map compiled in 1995 by the National Soil Survey Office and the SOC data provided by the same organisation (Fig. 5b). The SOC density data on this map were collected from 1979 to 1985 during the 2^nd^ China national soil survey, and they are the most recent soil data covering the entire area of China, which are still widely used in many recent literature^4^,^51^,^52^.

### Geographical Information Systems-based methodology for estimating carbon storage changes

The proposed methodology relies on the differences of carbon densities between land use types. Vegetation carbon and SOC densities differ between various land use types^1^. When one land use type is converted to another, its carbon density gradually gets close to the new land use type^15^. These theories made the calculations possible. Vegetation carbon and SOC densities are variable and can be influenced by many factors. In this paper, we only considered the differences of vegetation carbon and SOC densities between land use types and provincial regions. Influencing factors, such as climate, fertiliser, temperature and land management technique, were ignored. Because this study relies on mean density values, a thorough consideration of all the influencing factors and fluctuations may be unnecessary. With Geographical Information Systems (GIS) methods such as Combine Calculation and Zonal Statistics, this paper was able to implement the estimation of carbon storage changes from LUTC for each provincial-level region. The national values were calculated using the provincial results. Details of the processes are given in the following sections. To get finer results, the calculations were done using the 25 second-level land use types. Then, the results were summed or averaged according to the reclassified first-level types. However, to explain the basic idea of this methodology more clearly, we explained the process as if calculations had been done using the first-level types.

### Data preprocessing

Since data used in this paper came from different sources, these data were first projected to the same coordinate system. As preparation for the following analyses, other maps were cut into provincial-level extents using masks obtained from the provincial zoning map. This allowed us to obtain a set of data for each provincial-level region studied. After that, we dealt with the data abnormality in Xinjiang during the first period using the land use map of 2000 provided by the same organization. Then a quality assessment was conducted, which indicated that the quality of these data was sufficient for this research and that they could be used together in the ArcGIS9.3 software.

### Calculation of the land use transition matrices

The land use transition matrices were calculated using Combine Calculation. Combine Calculation is a simple but useful GIS method that can help differentiate the earlier and later land use combinations. Using the Raster Calculator function of the ArcGIS9.3 software, we applied Combine Calculation with the provincial land use maps of 1980 and 1995, and formed the land use transition matrix from 1980 to 1995 for each provincial-level region. With the provincial land use maps of 1995 and 2010, the same method was used to construct the land use transition matrices from 1995 to 2010. Then we summed the provincial values to calculate the national values. To better monitor the flows of LUTC, we summed the matrices by the out and in land use types, separately.

### Calculation of carbon densities for different land use types

Using the provincial SOC density maps and land use maps of the corresponding period (provincial land use maps of 1980), the average SOC density values of different land use types were calculated for each provincial region using the Zonal Statistics function of ArcGIS9.3. SOC density maps were the value data set, and land use maps were the zone data set. The Zonal Statistics function worked as follows (Equation (1)):


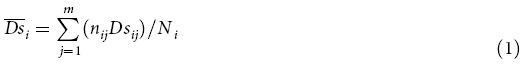


Where 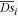 is the mean SOC density of land use type *i*, *n*_*ij*_ is the number of pixels of soil type *j* within the area of land use type *i*, *Ds*_*ij*_ is the carbon density of soil type *j* within the area of land use type *i*, *m* is the number of soil types within the area of land use type *i*, and *N*_*i*_ is the number of pixels for land use type *i*.

Using the same method, the mean vegetation carbon density values of different land use types were calculated for each provincial-level region using the provincial vegetation carbon density maps and land use maps of the corresponding period (provincial land use maps of 1980) (Equation (2)).


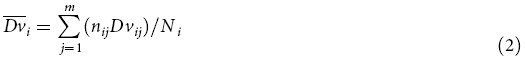


Where the variables’ meanings correspond to those in Equation (1).

To increase the understanding of the carbon density variations across China, we calculated the comprehensive carbon densities of different land use types by summing the vegetation carbon and SOC densities.

### Carbon storage changes caused by LUTC

Carbon storage changes due to LUTC were estimated using the difference between the carbon storages in terrestrial ecosystems at two points in time. We assumed that the average provincial SOC and vegetation carbon densities of the same land use type remain constant over the period studied. Based on this assumption, we first calculated the SOC storage changes for every provincial region during the period 1980-1995 as follows (Equation (3) & Equation (4)).


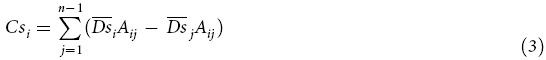



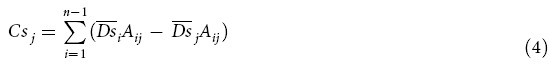


Where *Cs*_*i*_ is the provincial SOC storage change caused by the transfer out of land use type *i*, *Cs*_*j*_ is the provincial SOC storage change caused by the transfer in of land use type *j*, *n* is the number of land use types studied in this paper, 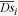 and 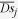 are the mean SOC densities of land use types *i* and *j*, *A*_*ij*_ is the area converted from land use type *i* to land use type *j* during the period 1980-1995.

Then the same calculations were done for the period 1995-2010, using the land use transition matrices from 1995 to 2010 calculated in the fourth part of the Methods section and the same SOC densities of different land use types.

Next, we calculated the carbon storage changes of the vegetation in the same way (Equation (5) & Equation (6)).


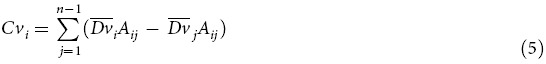



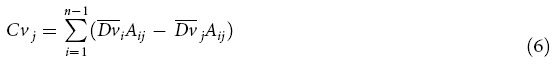


Where the variables’ meanings correspond to those in Equation (3) and Equation (4).

Then, we calculated the provincial composite carbon storage variations for the two periods using Equation (7) and Equation (8).


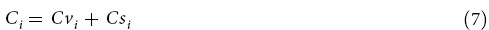



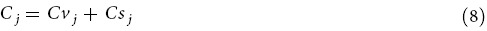


Where *C*_*i*_, *Cv*_*i*_ and *Cs*_*i*_ are the total, vegetation and soil carbon storage changes caused by the transfer out of land use type i in each region, and *C*_*j*_, *Cv*_*j*_ and *Cs*_*j*_ are the total, vegetation and soil carbon storage changes caused by the transfer in of land use type j in every region.

In the end, we summed the provincial carbon storage variations to produce the national values.

## Author Contributions

X.H. initiated the concept of the study. X.H. and M.Z. designed the research. M.Z. conducted the analyses and drafted the manuscript. X.C., L.L., J.T. and H.Y. provided strategic advices and comments on the manuscript. M.Z. and H.Y. revised the manuscript. All authors reviewed the manuscript.

## Additional Information

**How to cite this article**: Zhang, M. *et al.* Impact of land use type conversion on carbon storage in terrestrial ecosystems of China: A spatial-temporal perspective. *Sci. Rep.* **5**, 10233; doi: 10.1038/srep10233 (2015).

## Figures and Tables

**Figure 1:**
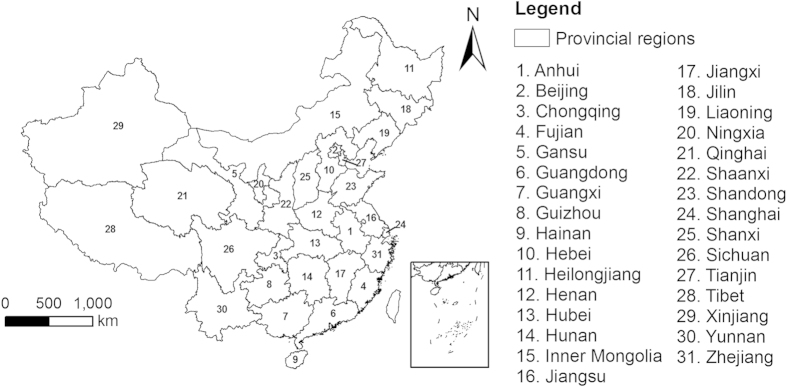
Map of the study area. Map created using ArcGIS 9.3 software. (Environmental Systems Research Institute (ESRI), Redlands, CA, USA).

**Table 1:** Carbon storage variations caused by LUTC in China (Positive values represent carbon loss; negative values represent carbon gain).

		Carbon storage variations of terrestrial ecosystems (Unit: Tg-C)			
Land use types		From transfer out of land use types during 1980 to 1995	From transfer in of land use types during 1980 to 1995	From transfer out of land use types during 1995 to 2010	From transfer in of land use types during 1995 to 2010
Cropland	**Total**	**−334**	**335**	**−415**	**499**
	Vegetation	−59	68	−73	96
	Soil	−275	267	−342	403
Forest land	**Total**	**942**	**−946**	**1340**	**−1398**
	Vegetation	229	−235	326	−308
	Soil	713	−711	1014	−1090
Grassland	**Total**	**632**	**−379**	**661**	**−863**
	Vegetation	−98	99	−107	105
	Soil	730	−478	768	−968
Wetland	**Total**	**−35**	**18**	**17**	**113**
	Vegetation	−10	9	−14	36
	Soil	−25	9	31	77
Settlements	**Total**	**−42**	**43**	**−50**	**96**
	Vegetation	−8	9	−8	19
	Soil	−34	34	−42	77
Other land	**Total**	**−944**	**1149**	**−1493**	**1613**
	Vegetation	−59	45	−68	108
	Soil	−885	1104	−1425	1505
Total	**Total**	**219**	**219**	**60**	**60**
	Vegetation	−6	−6	56	56
	Soil	225	225	4	4

**Table 2:** Key provincial-level regions for carbon storage loss during LUTC.

	Key regions and their carbon storage decreases caused by LUTC (Unit: Tg-C)			
Land use types	From transfer out of land use types during 1980 to 1995	From transfer in of land use types during 1980 to 1995	From transfer out of land use types during 1995 to 2010	From transfer in of land use types during 1995 to 2010
Cropland	Liaoning 8	Heilongjiang 112	Liaoning 9	Heilongjiang 143
	Tibet 8	Sichuan 84	Xinjiang 7	Sichuan 142
			Henan 7	
Forest land	Inner Mongolia 277		Inner Mongolia 346	
	Tibet 165		Heilongjiang 246	
	Heilongjiang 110		Tibet 213	
Grassland	Tibet 518	Inner Mongolia 175	Xinjiang 596	Inner Mongolia 203
			Qinghai 338	
Wetland	Inner Mongolia 16	Tibet 15	Xinjiang 22	Tibet 71
	Qinghai 9	Qinghai 7	Inner Mongolia 17	
Settlements		Henan 5		Heilongjiang 11
		Liaoning 4		Henan 9
		Anhui 4		Xinjiang 7
		Hubei 4		Liaoning 7
		Jilin 3		Anhui 7
		Jiangsu 3		Fujian 6
				Hubei 6
Other land		Tibet 678		Xinjiang 661
				Tibet 355

**Table 3:** Key provincial-level regions for carbon storage gain during LUTC.

	Key regions and their carbon storage increases caused by LUTC (Unit: Tg-C)			
Land use types	From transfer out of land use types during 1980 to 1995	From transfer in of land use types during 1980 to 1995	From transfer out of land use types during 1995 to 2010	From transfer in of land use types during 1995 to 2010
Cropland	Sichuan 86	Inner Mongolia 16	Sichuan 146	Xinjiang 15
	Heilongjiang 46	Xinjiang 12	Heilongjiang 99	Liaoning 10
	Gansu 44			
	Yunnan 23			
Forest land		Inner Mongolia 253		Inner Mongolia 534
		Tibet 208		Tibet 178
		Sichuan 66		
Grassland	Inner Mongolia 220	Qinghai 284	Inner Mongolia 415	Tibet 519
		Tibet 205		Qinghai 303
Wetland	Tibet 58	Inner Mongolia 14	Tibet 15	Qinghai 10
			Jilin 4	
Settlements	Henan 6		Heilongjiang 7	
	Anhui 4		Henan 6	
	Liaoning 4		Xinjiang 5	
	Hubei 3		Liaoning 4	
	Jilin 3		Anhui 4	
	Xinjiang 3			
Other land	Tibet 359		Tibet 686	
	Qinghai 289		Xinjiang 339	
